# MicroRNA‐365 modulates astrocyte conversion into neuron in adult rat brain after stroke by targeting *Pax6*

**DOI:** 10.1002/glia.23308

**Published:** 2018-02-16

**Authors:** Jia‐Lin Mo, Qi Liu, Zeng‐Wei Kou, Kun‐Wei Wu, Ping Yang, Xian‐Hua Chen, Feng‐Yan Sun

**Affiliations:** ^1^ Department of Neurobiology and State Key Laboratory of Medical Neurobiology, School of Basic Medical Sciences Shanghai Medical College, Fudan University Shanghai 200032 China; ^2^ Shanghai Key Laboratory of Clinical Geriatric Medicine Research Center on Aging and Medicine, Shanghai Medical College, Fudan University Shanghai 200032 China; ^3^ Institute for Basic Research on Aging and Medicine, School of Basic Medical Sciences Shanghai Medical College, Fudan University Shanghai 200032 China

**Keywords:** astrocyte, brain repair, glia, ischemic injury, microRNA, neurogenesis, neuron

## Abstract

Reactive astrocytes induced by ischemia can transdifferentiate into mature neurons. This neurogenic potential of astrocytes may have therapeutic value for brain injury. Epigenetic modifications are widely known to involve in developmental and adult neurogenesis. PAX6, a neurogenic fate determinant, contributes to the astrocyte‐to‐neuron conversion. However, it is unclear whether microRNAs (miRs) modulate PAX6‐mediated astrocyte‐to‐neuron conversion. In the present study we used bioinformatic approaches to predict miRs potentially targeting *Pax6*, and transient middle cerebral artery occlusion (MCAO) to model cerebral ischemic injury in adult rats. These rats were given striatal injection of glial fibrillary acidic protein targeted enhanced green fluorescence protein lentiviral vectors (Lv‐GFAP‐EGFP) to permit cell fate mapping for tracing astrocytes‐derived neurons. We verified that miR‐365 directly targets to the 3′‐UTR of *Pax6* by luciferase assay. We found that miR‐365 expression was significantly increased in the ischemic brain. Intraventricular injection of miR‐365 antagomir effectively increased astrocytic PAX6 expression and the number of new mature neurons derived from astrocytes in the ischemic striatum, and reduced neurological deficits as well as cerebral infarct volume. Conversely, miR‐365 agomir reduced PAX6 expression and neurogenesis, and worsened brain injury. Moreover, exogenous overexpression of PAX6 enhanced the astrocyte‐to‐neuron conversion and abolished the effects of miR‐365. Our results demonstrate that increase of miR‐365 in the ischemic brain inhibits astrocyte‐to‐neuron conversion by targeting *Pax6*, whereas knockdown of miR‐365 enhances PAX6‐mediated neurogenesis from astrocytes and attenuates neuronal injury in the brain after ischemic stroke. Our findings provide a foundation for developing novel therapeutic strategies for brain injury.

## 1 INTRODUCTION

Ischemic stroke induces neurogenesis in both neurogenic and non‐neurogenic brain regions (Arvidsson, Collin, Kirik, Kokaia, & Lindvall, 2002; Darsalia, Heldmann, Lindvall, & Kokaia, 2005; Jin et al., 2006; Nadareishvili & Hallenbeck, 2003; Parent, Vexler, Gong, Derugin, & Ferriero, 2002). These newly generated neurons can facilitate brain repair by integrating into local and distal neural networks (Hou et al., 2008; Sun et al., 2012; Wang et al., 2009; Zhang, Deng, Sun, Xu, & Sun, 2013). Recent studies have demonstrated that reactive astrocytes induced by ischemia exhibit properties of neural stem cells and can transdifferentiate into neurons (Magnusson et al., 2014; Shimada, LeComte, Granger, Quinlan, & Spees, 2012; Sirko et al., 2013). Moreover, similar to newborn neurons from the subgranular and subventricular zones, astrocytes‐derived neurons possess the characteristics of morphological and functional mature neurons (Duan et al., 2015; Guo et al., 2014), and reform neural circuitry with preexisting neurons in the ischemic regions of adult rat brain (Duan et al., 2015). These observations indicate that neurogenesis from ischemia‐induced reactive astrocytes might play important roles in neural repair following ischemic brain injury.

PAX6, a key transcription factor in the generation of neuronal lineages in the central nervous system (CNS; Hack et al., 2005; Hack, Sugimori, Lundberg, Nakafuku, & Gotz, 2004; Kallur, Gisler, Lindvall, & Kokaia, 2008; Manuel, Mi, Mason, & Price, 2015), can direct the astrocyte‐to‐neuron conversion. For example, forced expression of PAX6 induces cultured astrocytes conversion into neurons (Heins et al., 2002). Additionally, PAX6 overexpression causes reactive astrocytes to express the early neuronal markers in the cortex of mouse with a cortical stab wound lesion (Buffo et al., 2005) and transdifferentiate into neuroblasts in the ischemic brain (Kronenberg et al., 2010). These studies suggest that upregulation of PAX6 might be beneficial for promoting astrocyte‐to‐neuron conversion in the brain after ischemic injury.

MicroRNAs (miRs) are small (∼22 nt) non‐coding RNAs that bind to the 3′‐UTR of target mRNA and result in mRNA degradation or translational repression. MiRs participate in a wide variety of cellular processes, such as cell proliferation, differentiation, metabolism, and fate determination (Bartel, 2007; Carthew & Sontheimer, 2009; Li & Jin, 2010; Singh, 2007). Moreover, miRs mediate reprogramming of somatic cells into neural stem cells or neurons (Yang et al., 2017). Under pathological condition, miRs alter in the ischemic brain (Bhalala, Srikanth, & Kessler, 2013; Wu, Zuo, & Ji, 2012; Zhou, Ding, & Gu, 2016) and modulate ischemia‐induced neurogenesis in adult rat brain via notch and sonic hedgehog signaling pathways (Liu, Chopp, Zhang, & Zhang, 2013). It has been reported that miRs regulate adult neurogenesis by targeting multiple transcription factors (Shibata, Nakao, Kiyonari, Abe, & Aizawa, 2011). Therefore, we proposed that miRs might regulate the conversion of astrocytes into neurons via directing PAX6 expression in the brain after ischemic stroke.

In the present study, we used bioinformatic approaches to identify miRs which have potential binding sites in the 3′‐UTR of *Pax6* and transient middle cerebral artery occlusion model to induce ischemic brain injury and striatal injection of lentiviral‐GFAP‐EGFP vectors combined with cell fate mapping to trace the transition of astrocytes into neurons. We mainly found that miR‐365 inhibited ischemia‐induced astrocyte‐to‐neuron conversion by targeting *Pax6* and knockdown of miR‐365 enhanced the PAX6‐mediated astrocytes‐derived neurogenesis in the rat brain after ischemic injury.

## 2 MATERIALS AND METHODS

### 2.1 Prediction and synthesis of miRs targeting *Pax6*

MiRs targeting the 3′‐UTR of *Pax6* were predicted using TargetScan, miRDB and http://microRNA.org. Candidate miRs were selected based on their prediction by all three bioinformatic tools, and were conserved in human, mouse and rat. MiR agomir and antagomir oligonucleotides were synthesized and purified with high‐performance liquid chromatography by Shanghai GenePharma Co. Ltd. (Shanghai, China). All miR oligonucleotides were modified with 2′‐OMe and cholesterol. The sequences of the miRs are as follows: miR‐365 agomir (sense: 5′‐UAAUGCCCCUAAAAAUCCUUAU‐3′; antisense: 5′‐AAGGAUUUUUAGGGG CAUUAUU‐3′); miR‐7 agomir (sense: 5′‐UGGAAGACUAGUGAUUUUGUUGU‐3′; antisense: 5′‐AACAAAAUCACUAGUCUUCCAUU‐3′); miR‐129 agomir (sense: 5′‐CUUUUUGCGGUCUGGGCUUGC‐3′; antisense: 5′‐AAGCCCAGACCGC AAAAAGUU‐3′); miR agomir negative control (sense: 5′‐UUCUCCGAACGU GUCACGUTT‐3′; antisense: 5′‐ACGUGACACGUUCGGAGAATT‐3′); miR‐365 antagomir (5′‐AUAAGGAUUUUUAGGGGCAUUA‐3′); miR antagomir negative control (5′‐CAGUACUUUUGUGUAGUACAA‐3′).

### 2.2 Primary cortical astrocyte culture and miR transfection

Cortical astrocytes were obtained from newborn Sprague Dawley rats (1–3 days old). Briefly, after removal of blood vessels and pia mater, cerebral cortices were digested with 0.25% trypsin (Gibco, Rockville, MD, USA) at 37°C for 10 min, and dissociated cortical cells were suspended in DMEM (Gibco, Rockville, MD, USA) with 10% fetal bovine serum (FBS; Biological Industries, Cromwell, CT, USA). The cells were then passed through a 70 μm mesh (BD Biosciences, USA) and plated at a density of 1 × 10^6^ cells/ml on poly‐l‐lysine (0.1 mg/ml; Sigma‐Aldrich, St. Louis, MO, USA)‐coated dishes in high‐glucose DMEM with 10% FBS and antibiotics (100 U/ml penicillin + 100 μg/ml streptomycin; Gibco, Rockville, MD, USA) in a 5% CO_2_ humidified incubator at 37°C. The culture medium was changed every 3 days. Cells were passaged upon reaching 90% confluence. Astrocytes were identified with glial fibrillary acidic protein (GFAP) immunolabeling and were used for experiments when the number of GFAP‐positive cells exceeded 95% of the total. Cultured astrocytes were transfected with 50 nM agomir or antagomir or negative‐control (GenePharma; Ma et al., 2016) using lipofectamine 2000 (Invitrogen, Rockville, MD, USA) according to the manufacturer's protocol. The cells were harvested at 48 hr after transfection for further detection of miRs, mRNAs and proteins.

### 2.3 qRT‐PCR analysis

MiRs and mRNAs were extracted using the miRcute miR extraction kit (Tiangen) and Trizol reagent (Invitrogen) according to the manufacturers’ protocols. Reverse transcription of miR was performed using the One Step miR cDNA Synthesis Kit (HaiGene, Haerbin, China), and reverse transcription of mRNA was performed using the Golden^1st^ cDNA Synthesis Kit (HaiGene). PCR amplification was performed on the Eppendorf Mastercycler ep realplex (Eppendorf UK Limited, UK) using the Golden HS SYBR Green qPCR Mix Kit (HaiGene). MiR and mRNA levels were normalized to RNU6B small RNA and actin, respectively. Comparisons were calculated as the reverse log of the ΔΔCT from controls (Pfaffl, 2001). All assays were performed in triplicate. The primers used for qRT‐PCR were synthesized by Shanghai Sangon Biotech (Shanghai, China), and their sequences are as follows: *Pax6* (Forward: 5′‐CTGGAGAAAGAGTTTGAGAGGAC‐3′; Reverse: 5′‐GCTGTGGAATTGGCTGGTAG‐3′); *Actin* (Forward: 5′‐GGAGATTACTG CCCTGGCTCCTA‐3′; Reverse: 5′‐GACTCATCGTACTCCTGCTTGCTG‐3′); Rno‐miR‐365 (Forward: 5′‐GCAGTAATGCCCCTAAAAATCC‐3′; Reverse: 5′‐CAGGTCCAGTTTTTTTTTTTTTTTATAAG‐3′); Rno‐miR‐7 (Forward: 5′‐CGCAGTGGAAGACTAGTGAT‐3′; Reverse: 5′‐GTCCAGTTTTTTTTTTTTTTTACAAC‐3′); Rno‐miR‐129 (Forward: 5′‐GCAGCTTTTTGCGGTCTGG‐3′; Reverse: 5′‐TCCAGTTTTTTTTTTTTTTTGCAAG‐3′). RNU6B primers were included in the Golden HS SYBR Green qPCR Mix Kit.

### 2.4 Oxygen and glucose deprivation

Oxygen and glucose deprivation (OGD) was induced as previously described (Wu, Kou, Mo, Deng, & Sun, 2016). Briefly, cultured astrocytes were washed and incubated in deoxygenated glucose‐free DMEM (Gibco, Rockville, MD, USA). The cultures were then transferred to an anaerobic chamber filled with a gas mixture of 95% N_2_/5% CO_2_ at 37°C for 4 hr. At the end of OGD treatment, the medium was replaced with normal medium, and the cultures were returned to a normal atmosphere. Control cells were cultured under normoxic conditions without OGD treatment. With this condition, we harvested the cells at 1, 6, 12, and 24 hr after OGD treatment for the detection of miRs and proteins.

### 2.5 Luciferase assay

The full‐length rat *Pax6* 3′‐UTR (762 nt) was cloned from cDNA from rat cultured astrocytes (forward primer [1]: 5′‐CCGCTCGAGAGAGAGAGAAAGAGAGAGAA TGTGA‐3′; reverse primer [2]: 5′‐TTTTCCTTTTGCGGCCGCTTTTCAAATATAA ATGAAATTAAC‐3′). To generate the mutant *Pax6* 3′‐UTR, we synthesized two mutant primers containing a point mutation in the seed region of the *Pax6* 3′‐UTR (primer 3: 5′‐ATAACATACATAAGGCGATTTACA‐3′; primer 4: 5′‐ATTTGTAAATCGCCT TATGTATG‐3′). We used primers 1 and 3 or 2 and 4 to amplify from primary rat astrocyte cDNA to acquire PCR products A or B, respectively. Then, we used primers 1 and 2 to amplify from a mixture of PCR products A and B to obtain the mutant sequence. The wild‐type or mutant 3′‐UTR of *Pax6* was then inserted into the *XhoI* and *NotI* sites downstream of the humanized renilla luciferase gene in the psiCHECK‐2 vector (Promega) and confirmed by DNA sequencing (BioSune, Shanghai, China).

293T cells were purchased from the Cell Bank of the Chinese Academy of Sciences and seeded onto 24‐well plates 1 day before transfection. The wells were 50%–70% confluent on the day of transfection and were cotransfected with 200 ng reporter vector carrying the *Pax6* wild‐type or mutant 3′‐UTR and 50 nM miR agomir or negative control per well using lipofectamine 2000 according to the manufacturer's protocol. At 48 hr after transfection, the cells were lysed, and extracted proteins were assayed using a dual luciferase reporter assay system, E1910 (Promega), according to the manufacturer's protocol. Results were expressed as relative luciferase activity, while renilla luciferase activity was normalized to firefly luciferase activity.

### 2.6 Animals

Male Sprague Dawley rats (230–260 g) were purchased from the Shanghai Experimental Animal Center of the Chinese Academy of Sciences. All animal care protocols and experiments were reviewed and approved by the Medical Experimental Animal Administrative Committee of Shanghai Medical College of Fudan University. All efforts were made to minimize animal suffering and reduce the number of animals used.

### 2.7 Stereotactic virus injection

Lenti‐GFAP‐EGFP virus (Lv‐GFAP‐EGFP; containing the astrocyte‐specific GFAP promoter; 1 × 10^8^ TU/ml; GeneChem, Shanghai China) and Lenti‐*Pax6*‐mCherry virus (Lv‐*Pax6*; containing the rat *Pax6* CDS; 2 × 10^8^ TU/ml; GeneChem) were used to label astrocytes and overexpress PAX6, respectively. Lenti‐mCherry virus (Lv‐mCherry; 1 × 10^9^ TU/ml; GeneChem) was used as the control for Lv‐*Pax6*. The injection volumes for Lv‐GFAP‐EGFP, Lv‐*Pax6* and Lv‐mCherry were 2, 2.5, and 0.5 μl, respectively. Seven days before subjecting to the operation of middle cerebral artery occlusion (MCAO), the same animals were injected with a viral suspension in the ipsilateral striatum (Bregma: AP, +1.0 mm; ML, +2.5 mm; DV, −4.0 mm). The injection rate was 0.19 μl/min, and the glass pipette was left in place for an additional 15 min before being withdrawn at a rate of 1 mm/min.

### 2.8 Operation of MCAO

Animals were anesthetized with 10% chloral hydrate (360 mg/kg, i.p.). Arterial blood pO_2_, pCO_2_ and pH were monitored using an i‐STAT Analyzer (Abbott Laboratories, Chicago, USA). The rectal temperature was maintained at 37°C ± 0.5°C using a temperature‐regulated heat lamp (Wang, Guo, Qiu, Feng, & Sun, 2007). Rats with physiological variables within normal ranges were subjected to a left MCAO as described previously (Zhang et al., 2006). Briefly, a 4‐0 nylon monofilament with a rounded tip was introduced into the lumen of the left external carotid artery and gently advanced into the internal carotid artery until a slight resistance was felt. The filament was left in place for 30 min and then withdrawn.

### 2.9 pGfa2‐EGFP plasmid and miR injection

The pGfa2‐EGFP plasmid was generated as described previously (Shen et al., 2016). Within 30 min of reperfusion after MCAO, stereotaxic injection of the plasmid was performed in deeply anesthetized rats placed in a stereotaxic frame. A 3 μl volume of the plasmid mixture (1 μl of 5 μg/μl plasmid, 1 μl of sterile saline and 1 μl of lipofectamine 2000) was stereotaxically delivered into the ipsilateral striatum (Bregma: AP, +1.0 mm; ML, +2.5 mm; DV, −4.0 mm). The injection rate was 0.19 μl/min, and the glass pipette was left in the place for an additional 15 min before being withdrawn at a rate of 1 mm/min. The miR agomir, antagomir and negative controls were diluted to a final concentration of 100 μM for intracerebroventricular infusion (Ge et al., 2014; Ma et al., 2016; Tao et al., 2015). MiR oligomers (5 μl) were then combined with lipofectamine 2000 (5 μl) in an RNase‐free PCR tube and incubated for at least 20 min at room temperature. The total injection volume of 10 μl was <5% of the 580 μl average cerebral spinal fluid volume in rats, and was therefore unlikely to cause intracranial hypertension (Lai, Smith, Lamm, & Hildebrandt, 1983). The mixture was injected into the contralateral lateral ventricle (Bregma: AP, −0.80 mm; ML, −1.4 mm; DV, −3.6 mm) immediately after pGfa2‐EGFP plasmid injection. The injection rate was 0.25 μl/min, and the glass pipette was left in place for an additional 15 min before being withdrawn at a rate of 1 mm/min.

### 2.10 Neurological score measurement

Neurological deficits were assessed and scored according to Longa's five‐point scale method (Longa, Weinstein, Carlson, & Cummins, 1989). Briefly, the following grading system was applied: grade 0, no observable neurological deficit; grade 1, failure to extend right forepaw fully; grade 2, circling to right; grade 3, falling to right; grade 4, unable to walk spontaneously. The average of neurological score for each group was used to express the severity of neurological deficits. The higher scores reflect the severer function deficits.

### 2.11 5′‐Bromodeoxyuridine (BrdU) injection

To detect newborn neurons, we intraperitoneally injected freshly prepared BrdU (Sigma‐Aldrich, St. Louis, MO, USA) in rats at a concentration of 50 mg/kg body weight once daily, 4–6 days after MCAO (Zhang et al., 2006).

### 2.12 Western blotting analysis

The protein samples were subjected to 10% sodium dodecyl sulfate polyacrylamide gel electrophoresis (SDS‐PAGE) and transferred onto polyvinylidene fluoride (PVDF) membranes (Bio‐Rad). Membranes were blocked with 10% fat‐free milk in Tris‐buffered saline containing 0.1% Tween‐20 (TBST) for 2 hr at room temperature, and then incubated with anti‐rabbit PAX6 antibody (1:1,000; catalog 42‐6600; Thermo Fisher Scientific, Waltham, MA, USA) in 2% bovine serum albumin (BSA; Amresco) at 4°C overnight. Then, after washing, the membranes were incubated with anti‐rabbit IgG‐horseradish peroxidase (HRP) at room temperature for 1 hr (1:3,000; catalog sc‐2004; Santa Cruz Biotechnology, Santa Cruz, CA, USA). Protein bands were visualized using the Western Lightning Plus‐ECL kit (PerkinElmer, Akron, OH, USA). Bands were normalized to β‐actin by stripping the membranes and reprobing with mouse monoclonal β‐actin primary antibody (1:10,000; catalog A5316; Sigma‐Aldrich) and anti‐mouse IgG‐HRP secondary antibody (1:3,000; catalog sc‐2005; Santa Cruz Biotechnology, Santa Cruz, CA, USA). Films were scanned and then analyzed with Image J software (Version 1.45 m, NIH).

### 2.13 Brain section preparation

Rats were anesthetized with 10% chloral hydrate and transcardially perfused with 0.9% saline solution followed by 4% paraformaldehyde dissolved in 0.1 M phosphate‐buffer (pH 7.4). The brains were removed, postfixed for 4 hr in 4% paraformaldehyde, and cryoprotected using a graded sucrose series (20% and 30%) until they sank. A freezing microtome (Model 820‐II; Leica, Germany) was used to cut 30‐μm‐thick coronal sections. Sections were stored at −20°C in a cryoprotectant solution for histological analysis.

### 2.14 Fluoro‐Jade B staining

Brain sections were dried and dipped in an 80% ethanol solution containing 1% sodium hydroxide for 5 min, 70% ethanol for 2 min, and 0.06% potassium permanganate for 10 min. After rinsing with distilled water, the sections were incubated with Fluoro‐Jade B (Millipore, Billerica, MA, USA) solution at concentration of 4 mg/L containing 0.1% acetic acid for 20 min (Zhang et al., 2006). The signals of Fluoro‐Jade B staining were detected at an excitation of 480 nm and an emission of 525 nm under a fluorescence microscope. Infarct area, contralateral hemisphere area and ipsilateral hemisphere area were measured using Image J software, and areas were multiplied by the distance between sections to obtain the respective volumes. Infarct volume was calculated as a percentage of the contralateral hemisphere volume, as described previously (Swanson et al., 1990).

### 2.15 Immunohistochemical staining

For single labeling, brain sections were incubated with anti‐rabbit PAX6 (1:200) overnight at 4°C, and then incubated with the corresponding biotinylated secondary antibody and avidin‐biotin‐peroxidase complex (Vector Laboratories Inc., Burlingame, CA, USA). Immunoreactivity was detected with 0.05% diaminobenzidine (DAB; Sigma‐Aldrich, St. Louis, MO, USA) in Tris‐HCl buffer (0.1 M, pH 7.6) containing 0.03% H_2_O_2_.

For double labeling, brain sections were incubated with anti‐rabbit PAX6 overnight at 4°C, and then with rabbit secondary antibody and avidin‐biotin‐peroxidase complex (Vector Laboratories Inc., Burlingame, CA, USA). Immunoreactivity was detected with the Vectastain ABC‐AP kit (Vector Laboratories Inc., Burlingame, CA). Then, brain sections were washed and incubated with anti‐mouse GFAP (1:200; catalog MS‐1376‐P; Thermo Fisher Scientific, Waltham, MA, USA) overnight at 4°C, and subsequently with the mouse secondary antibody and avidin‐biotin‐peroxidase complex, and detected using DAB. Incubation without primary antibody served as the negative control, and no positive signal was detected.

### 2.16 Immunofluorescence labeling and confocal microscopy

For double labeling of GFAP and PAX6, GFP and PAX6 or BrdU and NeuN, brain sections were incubated with anti‐mouse GFAP (1:200), anti‐goat GFP (1:500; catalog ab5450; Abcam, Cambridge, UK) or anti‐mouse BrdU antibody (1:200; catalog 11170376001; Roche, Mannheim, Germany) at 4°C overnight. The sections were then incubated with Alexa Fluor 488‐conjugated donkey anti‐mouse IgG (H + L) or donkey anti‐goat IgG (H + L; 1:1,000; catalog A21202 and A11055, respectively; Life Technologies, Carlsbad, CA, USA). The sections were subsequently incubated with anti‐rabbit PAX6 (1:200) or anti‐rabbit NeuN (1:200; catalog ABN78; Millipore, Billerica, MA, USA) overnight at 4°C, followed by Alexa Fluor 594‐conjugated donkey anti‐rabbit IgG (H + L; 1:1,000; catalog A21207; Life Technologies, Carlsbad, CA, USA).

For triple labeling of GFP, GFAP and NeuN, brain sections were incubated with anti‐rabbit NeuN (1:200) at 4°C overnight and then with Alexa Fluor 594‐conjugated donkey anti‐rabbit IgG (H + L; 1:1,000). Thereafter, the brain sections were incubated with anti‐goat GFP (1:500) at 4°C overnight, and subsequently with Alexa Fluor 488‐conjugated donkey anti‐goat IgG (H + L; 1:1,000). Finally, the sections were incubated with anti‐mouse GFAP (1:200) at 4°C overnight, followed by Alexa Fluor 647‐conjugated donkey anti‐mouse IgG (H + L; 1:1,000; catalog A31571; Life Technologies, Carlsbad, CA, USA). For nuclear labeling, sections were incubated with 4',6‐diamidino‐2‐phenylindole (DAPI) for 15 min at room temperature. Fluorescent signals were detected using a confocal laser scanning microscope (TCS SP8; Leica, Heidelberg, Germany) at excitation and emission wavelengths of 499 and 519 nm (Alexa Fluor 488), 591 and 618 nm (Alexa Fluor 594) or 650 and 670 nm (Alexa Fluor 647), respectively.

### 2.17 Cell counting

Serial sections (every 12th section between 1.0 and −0.20 mm from the Bregma) were used for stereological quantification of GFAP^+^/PAX6^+^, GFP^+^/PAX6^+^, GFP^+^/NeuN^+^, and BrdU^+^/NeuN^+^ cells in the striatum. GFAP^+^/PAX6^+^ and BrdU^+^/NeuN^+^ counting were performed using a light microscope (Q570IW; Leica, Germany) with a 20× objective lens and a confocal laser scanning microscope (TCS SP8; Leica, Germany) with a 40× objective lens in the five views of infarction border, respectively, and the number of immunoreactive cells was expressed as cells/mm^3^ for each rat brain. To count double‐immunolabeled GFP^+^/PAX6^+^and GFP^+^/NeuN^+^ cells, we carried out total cell counting in the five views of needle border under a confocal laser scanning microscope. Newly generated neural progenitor cells and mature neurons derived from reactive astrocytes in the ischemic striatum were calculated as the percentage of GFP^+^/PAX6^+^ and GFP^+^/NeuN^+^ cells over the total number of GFP^+^ cells, respectively.

### 2.18 Statistical analysis

Statistical analysis was performed using GraphPad Prism software (Version 6.0). Statistical significance was determined either by unpaired two‐tailed Student's *t* test for comparisons between two groups or by one‐way ANOVA with Tukey's post‐hoc test for multiple group comparisons. All experiments were repeated at least three times, and representative experiments are shown. All results are given as the means ± *SEM*. Results were considered statistically significant at a *p* value of <0.05.

## 3 RESULTS

### 3.1 MiR‐365 is upregulated in the ischemic brain and hypoxic cultured astrocytes

**Figure 1:**
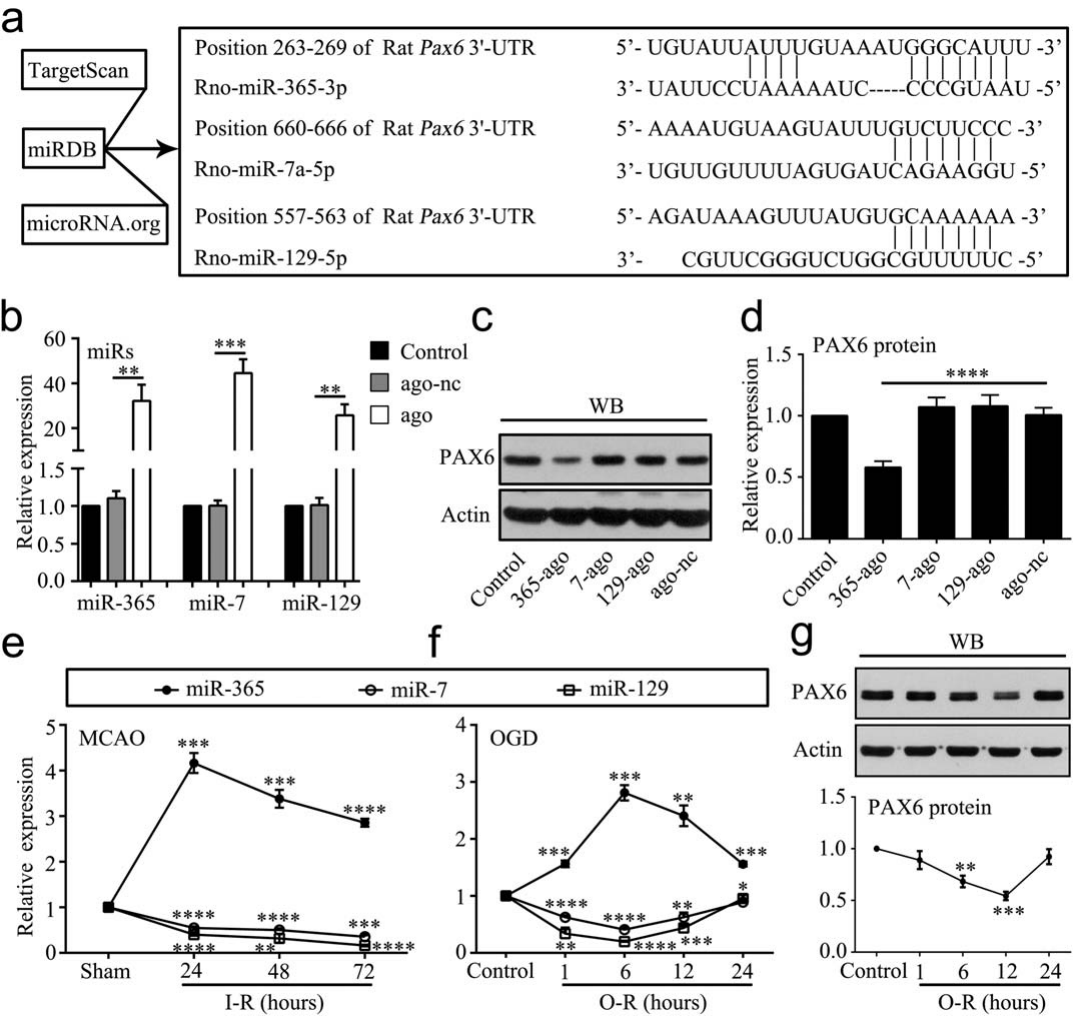
MiR‐365 is increased in the ischemic striatum and hypoxic cultured astrocytes. (a) Predicted miRs that have potential binding sites in the 3′‐UTR of *Pax6* using bioinformatics analysis. (b) qRT‐PCR analysis of miR‐365, miR‐7 and miR‐129 expression in cultured astrocytes 48 hr after transfection of their corresponding miR agomirs (ago) or agomir negative control (ago‐nc). All miR expression levels were normalized to endogenous control U6 snRNA and relative to control (*n *=* *3). (c and d) Expression levels of PAX6 protein in cultured astrocytes 48 hr after transfection of miR‐365‐ago (365‐ago), miR‐7‐ago (7‐ago), miR‐129‐ago (129‐ago), or ago‐nc were analyzed by WB. All protein expression levels were normalized to endogenous control actin and relative to control (*n *=* *5). (e) qRT‐PCR analysis of miR‐365, miR‐7 and miR‐129 expression in the ipsilateral striatum at 24, 48, and 72 hr after ischemia‐reperfusion (I‐R) (*n *=* *3). (f) qRT‐PCR analysis of miR‐365, miR‐7 and miR‐129 expression in cultured astrocytes at 1, 6, 12, and 24 hr after OGD‐reperfusion (O‐R) (*n *=* *3). (g) Expression levels of PAX6 protein in cultured astrocytes at 1, 6, 12, and 24 hr after O‐R were analyzed by western blotting (WB) (*n *=* *3). In e, f, and g, the *p* values are for comparisons versus the sham and control group, respectively; *p <* *.05, **p <* *.01, ***p <* *.001, and ****p <* *.0001 by unpaired two‐tailed Student's *t* test. In b and d, **p <* *.01, ***p <* *.001, and ****p <* *.0001 by one‐way ANOVA with Tukey's post‐hoc test. The data are presented as the means ± *SEM*

To investigate the regulation of *Pax6* by miRs, we used three bioinformatic programs (TargetScan, miRDB and http://microRNA.org) to identify miRs that potentially bind the 3′‐UTR of *Pax6*. MiR‐365‐3p (miR‐365), miR‐7a‐5p (miR‐7) and miR‐129‐5p (miR‐129) have potential binding sites in the 3′‐UTR of *Pax6* (Figure 1a). Next, we designed specific agomirs for miR‐365 (miR‐365‐ago), miR‐7 (miR‐7‐ago) and miR‐129 (miR‐129‐ago) to overexpress these miRs in cultured astrocytes (Figure 1b). We next assessed the effect of each miR on PAX6 protein expression. MiR‐365‐ago significantly inhibited PAX6 expression, while miR‐7‐ago and miR‐129‐ago had no effect on PAX6 protein levels compared with agomir negative control (ago‐nc; Figure 1c,d).

Then, we examined the levels of these miRs in the ischemic rat striatum at several timepoints after MCAO. MiR‐365 was upregulated, while miR‐7 and miR‐129 were downregulated in the ipsilateral striatum of rats at 24, 48, and 72 hr after MCAO compared with that in sham‐operated rats (Figure 1e). In addition, we performed an OGD‐reperfusion hypoxic treatment in cultured astrocytes. In consistent with the *in vivo* study, hypoxic treatment increased the levels of miR‐365, while it decreased the levels of miR‐7 and miR‐129 in cultured astrocytes at 1, 6, 12, and 24 hr after OGD‐reperfusion (Figure 1f). These results clearly indicate that ischemia causes miR‐365 upregulation. With the same condition, we examined the levels of PAX6 protein in cultured astrocytes. We found that OGD‐reperfusion treatment significantly reduced PAX6 expression in cultured astrocytes at 6 and 12 hr after hypoxic stimulation compared with control treatment (Figure 1g).

### 3.2 MiR‐365 directly modulates PAX6 expression in cultured astrocytes

**Figure 2:**
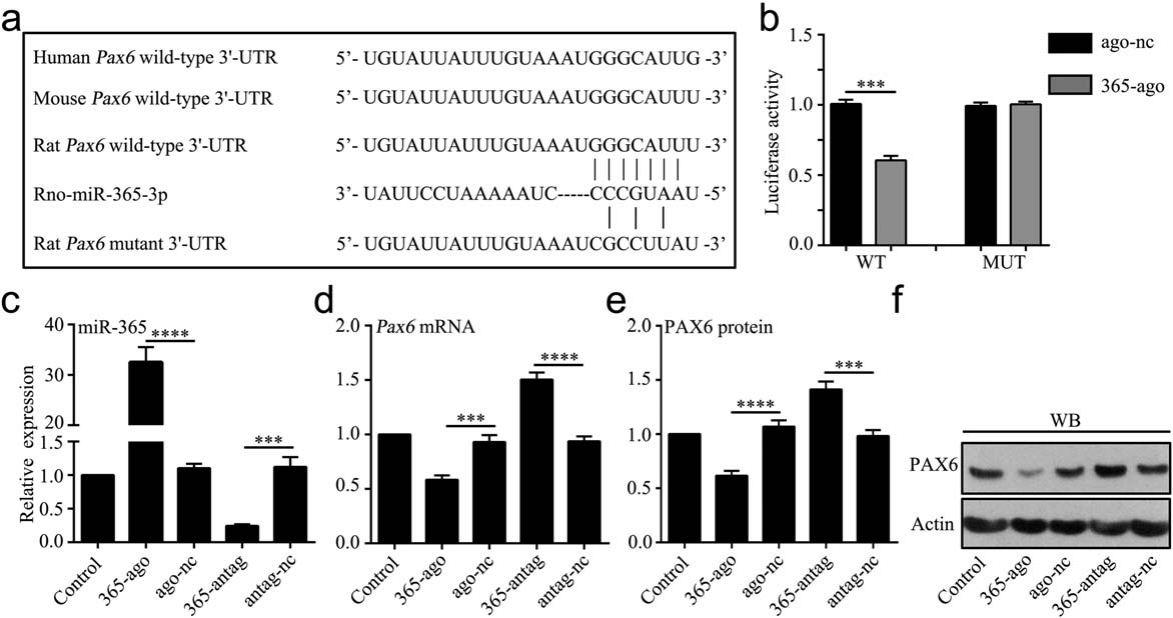
MiR‐365 directly targets PAX6 expression in cultured astrocytes. (a) Nucleotide sequence of the predicted miR‐365 binding site in the 3′‐UTR of *Pax6*. Shown are the seed sequence (CCCGUAA), wild‐type miR‐365 binding site (GGGCAUU) and the mutated miR‐365 binding site (CGCCUUA). (b) Luciferase assay in 293T cells 48 hr after transfection of luciferase reporter plasmid containing wild‐type (WT) or mutant (MUT) *Pax6* 3′‐UTR, together with 365‐ago or ago‐nc. Luciferase activity was calculated as renilla over firefly luciferase (*n *=* *3). (c) qRT‐PCR analysis of miR‐365 expression in cultured astrocytes 48 hr after transfection of 365‐ago, ago‐nc, miR‐365 antagomir (365‐antag) or antagomir negative control (antag‐nc) (*n *=* *3). (d–f) Expression levels of *Pax6* mRNA and PAX6 protein in cultured astrocytes 48 hr after different treatments were analyzed by qRT‐PCR and WB, respectively. All mRNA expression levels were normalized to endogenous control actin mRNA (*n *=* *5). In b, ***p <* *.001 by unpaired two‐tailed Student's *t* test; In c, d, and e, ***p <* *.001 and ****p <* *.0001 by one‐way ANOVA with Tukey's post‐hoc test. The data are presented as the means ± *SEM*

To assess whether miR‐365 directly targets the 3′‐UTR of *Pax6*, we cloned the wild‐type and mutant *Pax6* 3′‐UTR sequences into the pSiCheck vector to generate wild‐type (WT) and mutant (MUT) luciferase reporter plasmids, respectively (Figure 2a). We cotransfected with WT or MUT plasmids and miR‐365‐ago or ago‐nc in 293T cells and further detected luciferase activity at 48 hr after transfection. The results showed that the activity of WT luciferase was significantly decreased by miR‐365‐ago compared with ago‐nc. However, mutation of predicted target sites completely abolished the effects of miR‐365‐ago on reporter gene expression (Figure 2b). The results indicate that miR‐365 directly targets the 3′‐UTR of *Pax6*.

Next, we analyzed the expression of PAX6 in cultured astrocytes transfected with miR‐365‐ago, ago‐nc, miR‐365 antagomir (miR‐365‐antag) or antagomir negative control (antag‐nc) (Figure 2c). We found that miR‐365‐ago treatment reduced *Pax6* mRNA and protein levels compared with ago‐nc treatment. In contrast, both *Pax6* mRNA and protein were significantly increased in the miR‐365‐antag group compared with the antag‐nc group. Additionally, neither ago‐nc nor antag‐nc changed PAX6 expression compared with the controls (Figure 2d–f). These results suggest that miR‐365 directly affects PAX6 expression in the astrocytes.

### 3.3 MiR‐365 knockdown increases PAX6 expression in the astrocytes of rat brain after MCAO

**Figure 3:**
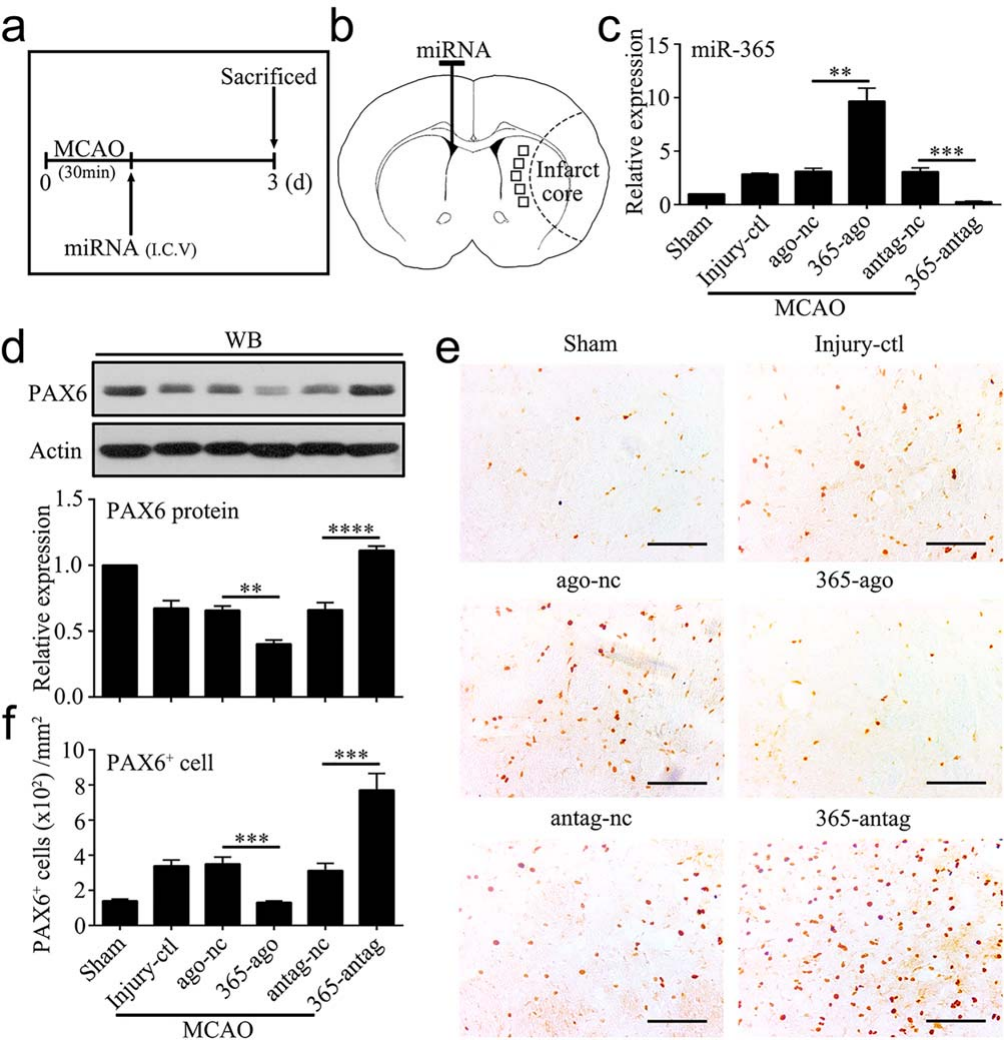
MiR‐365 antagomir increases PAX6 protein and PAX6‐positive cells in the ischemic striatum. (a) Illustration of animal experimental protocol. (b) Schematic of the brain showing the injection position of miRNA (contralateral ventricle) and the areas of immunoreactive cells counting (five fields of view). (c) qRT‐PCR analysis of miR‐365 expression in the ipsilateral striatum of rats 3 days after MCAO (*n *=* *3). Injury control rats (Injury‐ctl) indicated MCAO‐operated rats without other interventions. (d) Expression levels of PAX6 protein in the ipsilateral striatum of rats 3 days after MCAO were analyzed by Western blotting (WB) (*n *=* *5). (e) Representative images of immunolabeling of PAX6 in the brain sections of rats 3 days after MCAO. Scale bars: 100 μm. (f) The number of PAX6^+^ cell was counted as the average of the number of positive cells in the five fields of view (*n *=* *5). **p <* *.01, ***p <* *.001, and ****p <* *.0001 by one‐way ANOVA with Tukey's post‐hoc test. The data are presented as the means ± *SEM* [Color figure can be viewed at http://wileyonlinelibrary.com]

**Figure 4:**
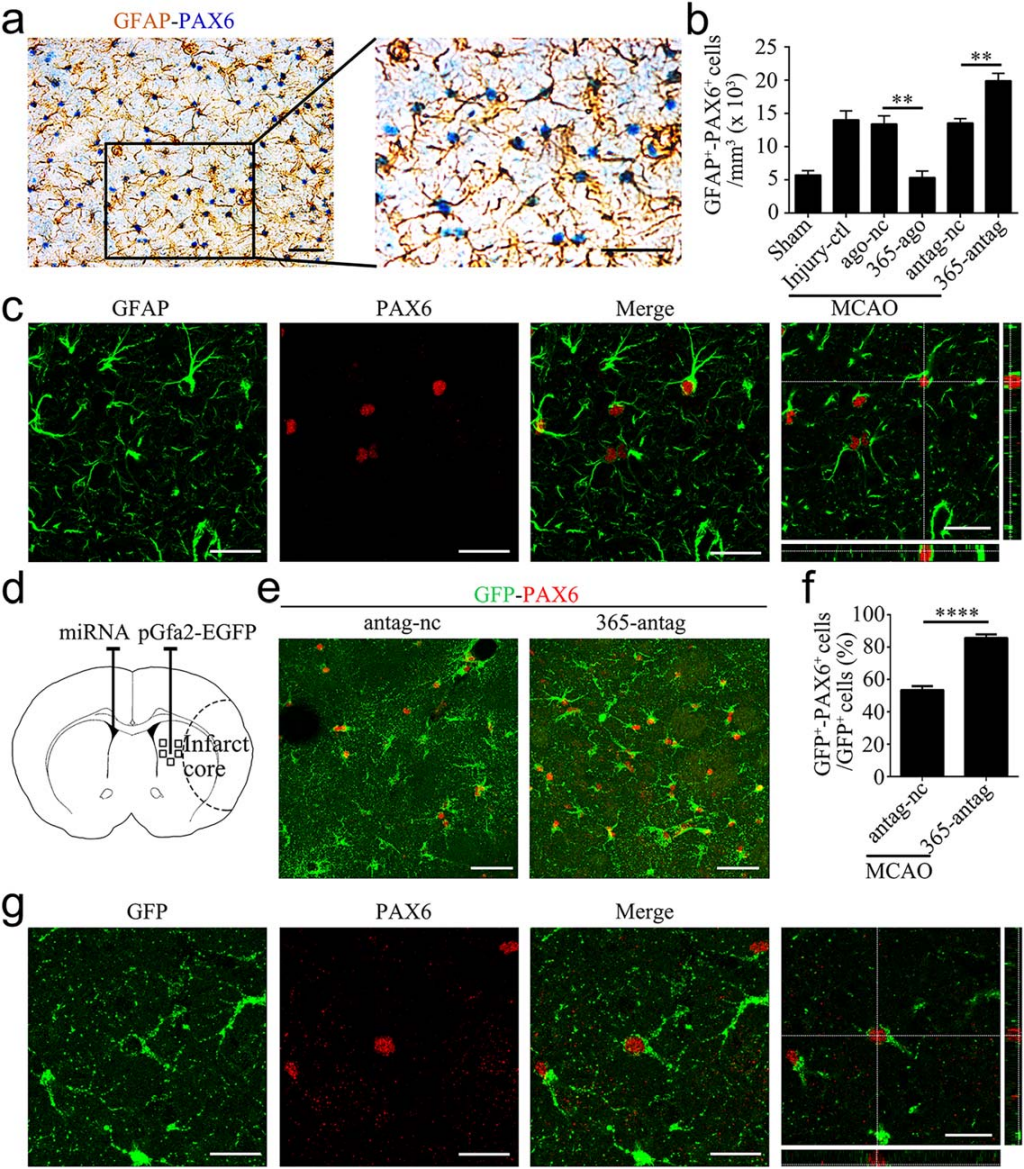
MiR‐365 antagomir increases PAX6‐expressing astrocytes in the ischemic striatum. (a) Representative images of immunolabeling of GFAP and PAX6 in the brain sections of rats 3 days after MCAO. Scale bars: 50 μm. (b) The number of GFAP^+^/PAX6^+^ (GFAP^+^‐PAX6^+^) cells was counted as the average of the number of positive cells in the five fields of view (Figure 3b; *n *=* *3 in the sham group; *n *=* *5 in the other groups). (c) Confocal microphotograph showing co‐labeling of GFAP and PAX6. Scale bars: 20 μm. (d) Schematic of the brain showing the injection position of pGfa2‐EGFP plasmid (ipsilateral striatum) and miRNA (contralateral ventricle) and the areas of immunoreactive cells counting (five fields of view). (e) Representative images of immunofluorescent double labeling of GFP and PAX6 in the brain sections of rats 3 days after MCAO. Scale bars: 50 μm. (f) The number of GFP^+^/PAX6^+^ cell was counted as the percentage of the total number of GFP^+^ cells in the five fields of view (*n *=* *7 in the antag‐nc group; *n *=* *8 in the 365‐antag group). (g) Confocal microphotograph showing co‐labeling of GFP and PAX6. Scale bars: 20 μm. In f, ****p <* *.0001 by unpaired two‐tailed Student's *t* test; In b, **p <* *.01 by one‐way ANOVA with Tukey's post‐hoc test. The data are presented as the means ± *SEM* [Color figure can be viewed at http://wileyonlinelibrary.com]

As described above, cerebral ischemia increased miR‐365 levels in the brain, and miR‐365 repressed PAX6 expression in the astrocytes. Therefore, we further analyzed the effects of miR‐365‐ago and miR‐365‐antag on the expression of PAX6 in the rat brain after MCAO. The rats were subjected to sham operation or a 30‐min period of MCAO (Injury‐ctl). Following MCAO, the rats were given contralateral ventricular injection of ago‐nc, miR‐365‐ago, antag‐nc or miR‐365‐antag, and sacrificed 3 days after MCAO (Figure 3a,b). We confirmed that miR‐365‐ago increased, while miR‐365‐antag decreased miR‐365 levels in the ipsilateral striatum compared with ago‐nc and antag‐nc, respectively (Figure 3c). With this experimental condition, we studied the effects of miR‐365‐ago and miR‐365‐antag on PAX6 protein level and PAX6 immunopositive (PAX6^+^) cell in the ischemic striatum using western blotting analysis and single immunohistochemical staining, respectively. The results showed that miR‐365‐ago treatment reduced the level of PAX6 protein (Figure 3d) and the number of PAX6^+^ cells compared with ago‐nc treatment (Figure 3e,f). In contrast, miR‐365‐antag treatment significantly increased PAX6 expression and the number of PAX6^+^ cells (Figure 3d–f). Then, we performed double immunostaining to determine colocalization of PAX6 and GFAP, an astrocytic marker, in the rat brain 3 days after MCAO. The results clearly showed that the PAX6^+^ staining was mainly detected in the GFAP positive astrocytes (GFAP^+^/PAX6^+^) as determined by double immunohistochemical (Figure 4a) and immunofluorescent images (Figure 4c). Moreover, the immunohistochemical results showed that miR‐365‐antag treatment significantly increased the number of GFAP^+^/PAX6^+^ cells compared with Injury‐ctl or antag‐nc treatment. Conversely, miR‐365‐ago treatment significantly reduced the number of GFAP^+^/PAX6^+^ cells compared with Injury‐ctl or ago‐nc treatment (Figure 4b). These results demonstrate that inhibition of miR‐365 by its antagomir elevates PAX6 expression in the astrocytes of ischemic brain.

Astrocytes activated by cerebral ischemic injury can be reprogrammed into neural precursor cells and transdifferentiate into functional mature neurons in adult mammalian brain (Duan et al., 2015), and PAX6 can direct astrocytes towards neuronal differentiation (Heins et al., 2002; Kronenberg et al., 2010). These observations combined with our present results show that miR‐365, highly upregulated in the ischemic brain, robustly inhibits PAX6 expression, suggesting that lowering miR‐365 expression might promote astrocytic reprogramming. To test this possibility, we performed ipsilateral striatal injection of a plasmid containing astrocyte‐specific GFAP promoter (pGfa2‐EGFP) to label reactive astrocytes and contralateral ventricular injection of miR‐365‐antag to downregulate miR‐365 immediately following MCAO (Figure 4d). Double immunolabeling for GFP and PAX6 (GFP^+^/PAX6^+^) in the brain sections of rats 3 days after MCAO revealed that miR‐365‐antag treatment significantly increased the percentage of GFP^+^/PAX6^+^ cells over the total number of GFP^+^ cells, compared with antag‐nc treatment (Figure 4e–g; 1014 GFP^+^/PAX6^+^ cells in 1181 GFP^+^ cells in the 365‐antag group; 566 GFP^+^/PAX6^+^ cells in 1048 GFP^+^ cells in the antag‐nc group). These results suggest that inhibition of miR‐365 enhances the reprogramming of reactive astrocytes in the ischemic brain by upregulating PAX6 expression.

### 3.4 MiR‐365 knockdown enhances conversion of astrocytes into mature neurons in rat brain after MCAO

**Figure 5:**
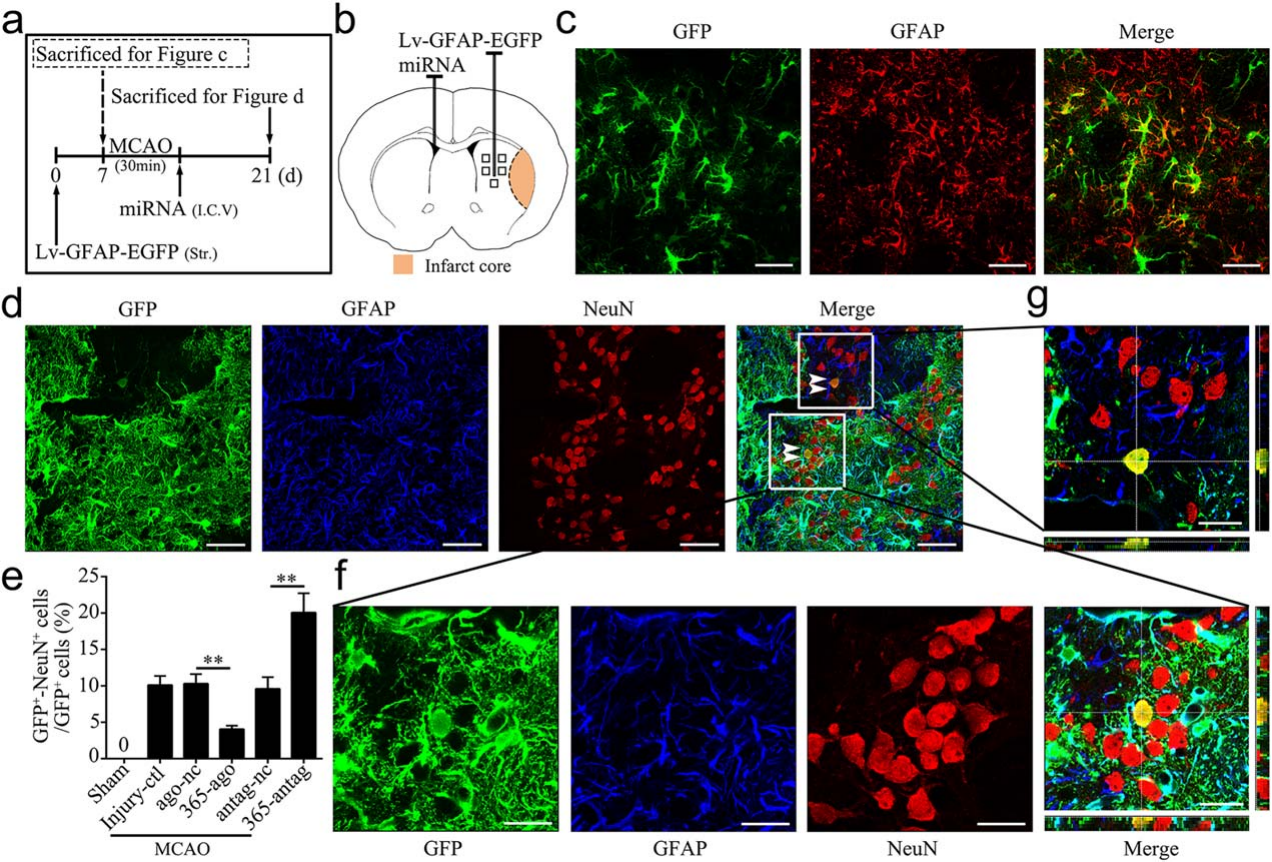
MiR‐365 antagomir increases the conversion of astrocytes into mature neurons in the ischemic striatum. (a) Illustration of animal experimental protocol. (b) Schematic of the brain showing the injection position of Lv‐GFAP‐EGFP (ipsilateral striatum) and miRNA (contralateral ventricle) and the areas of immunoreactive cells counting (five fields of view). (c) Representative images of immunofluorescent double labeling of GFP and GFAP in the brain sections of rats 7 days after Lv‐GFAP‐EGFP injection. Scale bars: 50 μm. (d) Representative images of immunofluorescent triple labeling of GFP and GFAP and NeuN in the brain sections of rats 14 days after MCAO. The white double arrowheads indicate GFP^+^/NeuN^+^ cells. Scale bars: 50 μm. (e) The number of GFP^+^/NeuN^+^ cell was counted as the percentage of the total number of GFP^+^ cells in the five fields of view (*n *=* *3 in the sham group; *n *=* *6 in the other groups). (f and g) Confocal microphotograph showing co‐labeling of GFP and NeuN. Scale bars: 20 μm. **p <* *.01 by one‐way ANOVA with Tukey's post‐hoc test. The data are presented as the means ± *SEM* [Color figure can be viewed at http://wileyonlinelibrary.com]

Our previous study has demonstrated that reactive astrocytes can transdifferentiate into different lineages of neurons, including neural stem/progenitor cells, immature and mature neurons. Beside, new neurons can develop into functional mature neurons and rebuild neural networks within the injured brain regions (Duan et al., 2015). In the present study we used a matured neuronal marker to trace reactive astrocyte neurogenic fate. With this model, we investigated the role of miR‐365 in the conversion of reactive astrocytes into mature neurons. As shown in Figure 5a,b, we injected Lv‐GFAP‐EGFP (a lentivirus with the astrocyte‐specific GFAP promoter) into the ipsilateral striatum of rats 7 days before MCAO, and miR‐365‐ago or miR‐365‐antag or negative controls into the contralateral ventricle immediately following MCAO. We then sacrificed the rats 14 days after initiating ischemia. First, we performed double immunolabeling for GFP and GFAP to confirm that Lv‐GFAP‐EGFP specifically labeled astrocytes in the normal brain (Figure 5c). Next, we performed immunofluorescent double labeling for GFP and NeuN. We found that 10.2% GFP^+^ cells were become mature neurons in the Injury‐ctl group (Figure 5d–g; 62 GFP^+^/NeuN^+^ cells in 610 GFP^+^ cells), although the majority of these cells still showed GFP^+^/GFAP^+^ astrocytes morphology. Moreover, miR‐365‐ago treatment significantly reduced the percentage of GFP^+^/NeuN^+^ cells over the total number of GFP^+^ cells compared with ago‐nc treatment or Injury‐ctl (Figure 5e; 26 GFP^+^/NeuN^+^ cells in 652 GFP^+^ cells in the 365‐ago group; 68 GFP^+^/NeuN^+^ cells in 683 GFP^+^ cells in the ago‐nc group). Interestingly, in the miR‐365‐antag‐treated rats, the percentage of GFP^+^/NeuN^+^ cell was significantly increased compared with antag‐nc‐treated or Injury‐ctl rats (Figure 5e; 142 GFP^+^/NeuN^+^ cells in 680 GFP^+^ cells in the 365‐antag group; 61 GFP^+^/NeuN^+^ cells in 628 GFP^+^ cells in the antag‐nc group). These results clearly demonstrate that endogenous miR‐365 inhibits the conversion of astrocytes into mature neurons in the rat brain after ischemic injury. Moreover, reducing endogenous miR‐365 expression promotes astrocyte‐to‐neuron conversion.

### 3.5 PAX6 overexpression abolishes the miR‐365‐mediated reduction of astrocyte‐to‐neuron conversion in rat brain after MCAO

**Figure 6:**
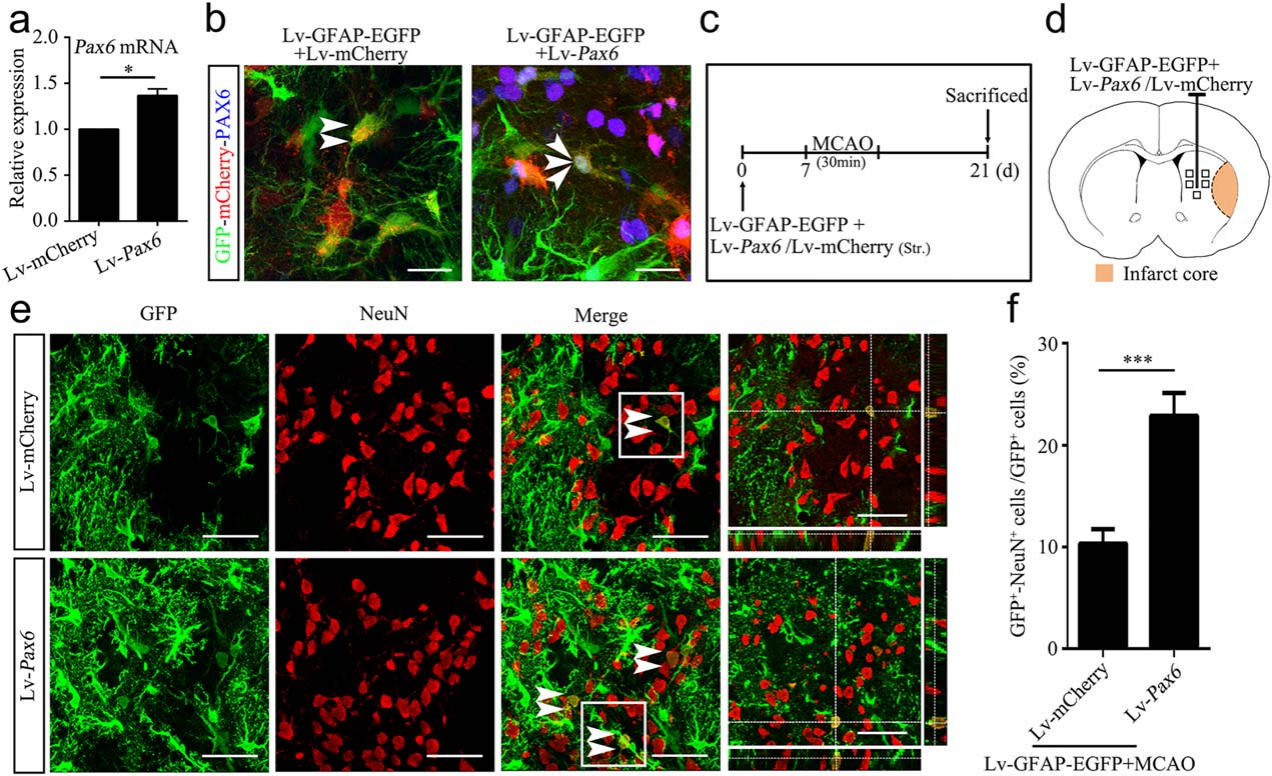
Overexpression of PAX6 increases the conversion of astrocytes into mature neurons in the ischemic striatum. (a) qRT‐PCR analysis of *Pax6* mRNA expression in the normal striatum of rats 7 days after Lv‐*Pax6* or Lv‐mCherry injection (*n *=* *3). (b) Representative images of immunofluorescent triple labeling of GFP and mCherry and PAX6 in the brain sections of rats 7 days after mixture lentivirus injection. The white double arrowheads indicate GFP^+^/mCherry^+^ cells, while the white triple arrowheads indicate GFP^+^/mCherry^+^/PAX6^+^ cells. Scale bars: 20 μm. (c) Illustration of animal experimental protocol. (d) Schematic of the brain showing the injection position of the mixture of Lv‐GFAP‐EGFP and Lv‐*Pax6* or Lv‐mCherry (ipsilateral striatum) and the areas of immunoreactive cells counting (five fields of view). (e) Representative images of immunofluorescent double labeling of GFP and NeuN in the brain sections of rats 14 days after MCAO. The white double arrowheads indicate GFP^+^/NeuN^+^ cells. Scale bars: 50 μm. (f) The number of GFP^+^/NeuN^+^ cell was counted as the percentage of the total number of GFP^+^ cells in the five fields of view (*n *=* *6). *p <* *.05 and ***p <* *.001 by unpaired two‐tailed Student's *t* test. The data are presented as the means ± *SEM* [Color figure can be viewed at http://wileyonlinelibrary.com]

In order to explore the role of PAX6 in the astrocyte‐neuron conversion, we constructed the lenti‐*Pax6*‐mCherry vector (Lv‐*Pax6*), containing the rat *Pax6* CDS without the 3′‐UTR, to upregulate PAX6 expression. We confirmed that Lv‐*Pax6* transduction caused higher *Pax6* mRNA expression, compared with Lv‐mCherry, in the normal brain (Figure 6a). We then injected a mixture of the Lv‐GFAP‐EGFP and Lv‐*Pax6* or Lv‐mCherry into the striatum of normal rats to assess whether exogenous PAX6 expressed in the astrocytes labeled for GFP. Immunolabeling of brain sections demonstrated that some GFP‐labeled astrocytes colocalized with mCherry and PAX6 in the Lv‐*Pax6* group, but only with mCherry in the Lv‐mCherry group (Figure 6b). Then, we injected a mixture of the Lv‐GFAP‐EGFP and Lv‐*Pax6* or Lv‐mCherry into the ipsilateral striatum of rats 7 days before MCAO and sacrificed the rats 14 days after the induction of ischemia to assess the effects of exogenous PAX6 on ischemia‐induced astrocyte‐to‐neuron conversion (Figure 6c,d). Newly generated mature neurons derived from reactive astrocytes, identified as GFP^+^/NeuN^+^ cells, were significantly increased in the Lv‐*Pax6* group, compared with that in the Lv‐mCherry group (Figure 6e,f; 132 GFP^+^/NeuN^+^ cells in 562 GFP^+^ cells in the Lv‐*Pax6* group; 66 GFP^+^/NeuN^+^ cells in 610 GFP^+^ cells in the Lv‐mCherry group), suggesting that enhanced expression of PAX6 in the ischemic brain promotes the conversion of astrocytes into mature neurons.

**Figure 7:**
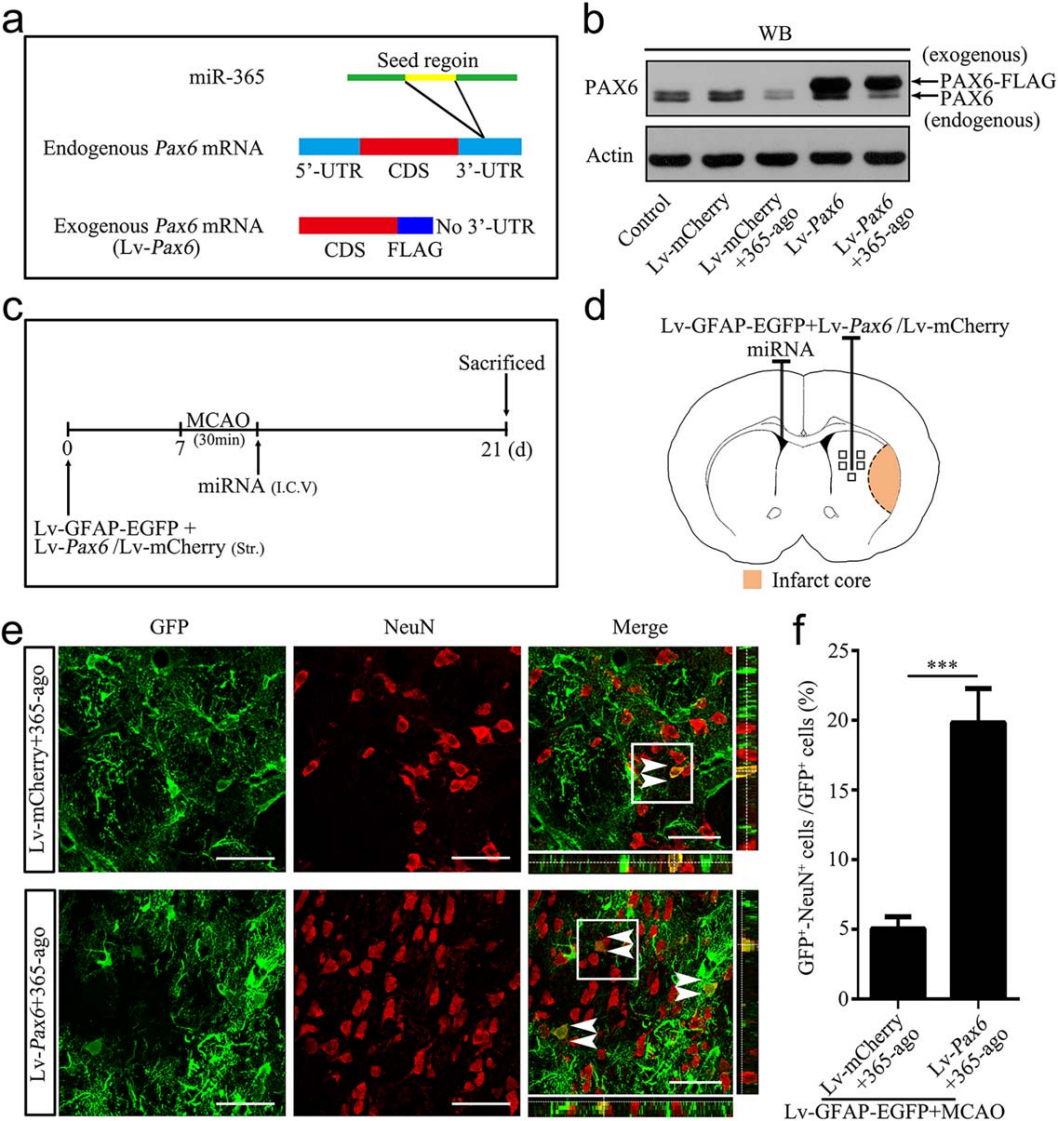
Overexpression of PAX6 abolishes the miR‐365‐mediated inhibition of astrocyte‐to‐neuron conversion in the ischemic striatum. (a) Illustration of the construction of the endogenous *Pax6* mRNA and the exogenous *Pax6* mRNA (Lv‐*Pax6*). (b) Expression levels of PAX6 protein in cultured astrocytes 7 days after Lv‐*Pax6* or Lv‐mCherry transfection, in combination with 365‐ago treatment, were analyzed by WB. (c) Illustration of animal experimental protocol. (d) Schematic of the brain showing the injection position of the mixture of Lv‐GFAP‐EGFP and Lv‐*Pax6* or Lv‐mCherry (ipsilateral striatum) and miRNA (contralateral ventricle) and the areas of immunoreactive cells counting (five fields of view). (e) Representative images of immunofluorescent double labeling of GFP and NeuN in the brain sections of rats 14 days after MCAO. The white double arrowheads indicate GFP^+^/NeuN^+^ cells. Scale bars: 50 μm. (f) The number of GFP^+^/NeuN^+^ cell was counted as the percentage of the total number of GFP^+^ cells in the five fields of view (*n *=* *6). ***p <* *.001 by unpaired two‐tailed Student's *t* test. The data are presented as the means ± *SEM* [Color figure can be viewed at http://wileyonlinelibrary.com]

To investigate whether the inhibitory effect of miR‐365 on astrocyte‐to‐neuron conversion involves a direct effect on *Pax6* expression, we overexpressed PAX6 by Lv‐*Pax6* injection in the brain in combination with miR‐365‐ago treatment. Theoretically, miR‐365 should not be able to inhibit the exogenous overexpressed PAX6 since its 3′‐UTR, a target region of miR‐365, was removed (Figure 7a). To determine this hypothesis, we treated cultured astrocytes with miR‐365‐ago and Lv‐*Pax6* (Lv‐*Pax6* + 365‐ago) or Lv‐mCherry (Lv‐mCherry + 365‐ago). The results showed that miR‐365‐ago inhibited endogenous PAX6 expression without affecting exogenous PAX6 expression (Figure 7b), suggesting that miR‐365 targets *Pax6* expression by directly binding to its 3′‐UTR.

Next, we performed ipsilateral striatal co‐injection of Lv‐GFAP‐EGFP and Lv‐*Pax6* or Lv‐mCherry 7 days before MCAO, combined with contralateral ventricular injection of miR‐365‐ago, immediately following MCAO. The rats were sacrificed 14 days after MCAO (Figure 7c,d). GFP^+^/NeuN^+^ cells were significantly increased in the Lv‐*Pax6* + miR‐365‐ago group, compared with that in the Lv‐mCherry + miR‐365‐ago group (Figure 7e,f; 119 GFP^+^/NeuN^+^ cells in 624 GFP^+^ cells in the Lv‐*Pax6* + 365‐ago group; 29 GFP^+^/NeuN^+^ cells in 572 GFP^+^ cells in the Lv‐mCherry + 365‐ago group), suggesting that exogenous overexpression of PAX6 abolishes the inhibitory effect of miR‐365 on the astrocyte‐to‐neuron conversion.

### 3.6 MiR‐365 knockdown enhances neurogenesis and reduces brain damage in rat after MCAO

**Figure 8:**
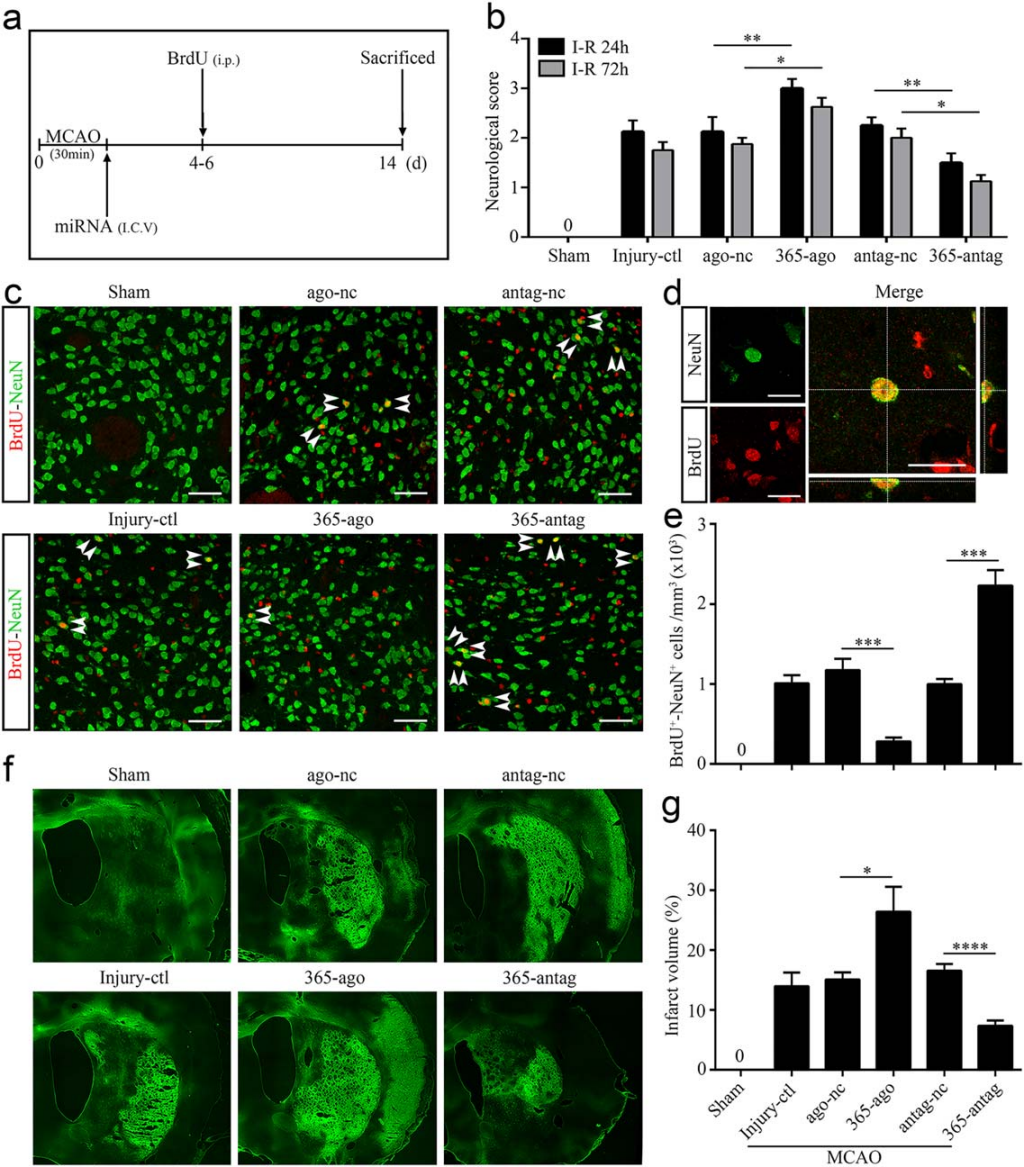
MiR‐365 antagomir promotes striatal neurogenesis and reduces brain damage after ischemic stroke. (a) Illustration of animal experimental protocol. (b) Neurological performance was assessed and scored according to Longa's method 1 and 3 days after MCAO (*n *=* *3 in the sham group; *n *=* *8 in the other groups). (c) Representative images of immunofluorescent double labeling of BrdU and NeuN in the brain sections of rats 14 days after MCAO. Scale bars: 50 μm. (d) Confocal microphotograph showing co‐labeling of BrdU and NeuN. Scale bars: 20 μm. (e) The number of NeuN^+^/BrdU^+^ cell was counted as the average of the number of positive cells in the five fields of view (Figure 3b; *n *=* *3 in the sham group; *n *=* *5 in the other groups). (f) Representative images of Fluoro‐Jade B staining in the brain sections of rats 14 days after MCAO. The bright green areas indicated infarct areas. (g) Infarct volume was calculated as a percentage of contralateral hemisphere volume (*n *=* *3 in the sham group; *n *=* *6 in the injury‐ctl, ago‐nc and 365‐ago groups; *n *=* *10 in the antag‐nc and 365‐antag groups). *p <* *.05, **p <* *.01, ***p <* *.001, and ****p <* *.0001 by one‐way ANOVA with Tukey's post‐hoc test. The data are presented as the means ± *SEM* [Color figure can be viewed at http://wileyonlinelibrary.com]

As mentioned above, inhibition of miR‐365 enhanced the stroke‐induced conversion of astrocytes into mature neurons. These new mature neurons derived from astrocytes can functionally integrate into neural networks (Duan et al., 2015), which might contribute to brain repair after ischemic injury. Therefore, we next tested the effects of miR‐365 on neurogenesis and brain repair after MCAO. The rats were subjected to sham operation or a 30‐min period of MCAO (Injury‐ctl). Following MCAO, the rats were given contralateral ventricular injection of ago‐nc, miR‐365‐ago, antag‐nc or miR‐365‐antag, and sacrificed 14 days after MCAO (Figure 8a). Meanwhile, we observed the effects of miR‐365 on neurological function in the rats after ischemic brain injury. The results showed that miR‐365‐ago treatment deteriorated neurological deficits compared with ago‐nc or vehicle (Injury‐ctl) treatment (Figure 8b). On the contrary, miR‐365‐antag treatment significantly reduced the severity of stroke‐induced neurological deficits compared with antag‐nc or vehicle (Injury‐ctl) treatment (Figure 8b).

Then, we performed double immunolabeling for BrdU and NeuN to label newly generated mature neurons in the brain sections of rats 14 days after MCAO. We found that miR‐365‐ago treatment significantly reduced the number of BrdU^+^/NeuN^+^ cells in the ischemic striatum compared with vehicle (Injury‐ctl) or ago‐nc treatment. However, miR‐365‐antag treatment dramatically increased the number of BrdU^+^/NeuN^+^ cells compared with antag‐nc treatment or Injury‐ctl (Figure 8c–e). In contrast, compared with that in the corresponding control or Injury‐ctl groups, infarct volume was increased in the miR‐365‐ago group and significantly reduced in the miR‐365‐antag group (Figure 8f,g). Taken together, these results indicate that miR‐365 worsens ischemic injury and suppresses neurogenesis in the brain after stroke. Interestingly, knockdown of miR‐365 enhances neurogenesis and reduces brain damage.

## 4 DISCUSSION

This study provides the first evidence that miR‐365, upregulated in the ischemic brain, inhibits the stroke‐induced conversion of reactive astrocytes into neurons via inhibition of PAX6 expression by targeting the 3′‐UTR of *Pax6*, and exacerbates ischemic brain injury. Interestingly, knockdown of miR‐365 with an antagomir increased PAX6 expression in the astrocytes and enhanced the astrocyte‐to‐neuron conversion, and reduced cerebral ischemic injury. These findings provided a foundation for further preclinical study to develop novel therapeutic targets for enhancement of brain repair.

Astrocytes, as a major type of neural cells in the mammalian brain, play vital roles in the CNS under physiological (Allen & Barres, 2009; Alvarez, Katayama, & Prat, 2013; Dong & Benveniste, 2001; Kirischuk, Heja, Kardos, & Billups, 2016; Liu, Ni, & Sun, 2017) and pathophysiological conditions (Buffo, Rolando, & Ceruti, 2010). For example, reactive astrocytes induced by ischemic injury can accelerate the formation of glial scars (Boda & Buffo, 2010). However, astrocytes also promote neuronal survival and neurogenesis by releasing various growth factors after ischemic stroke (Liu, Teschemacher, & Kasparov, 2017; Okoreeh, Bake, & Sohrabji, 2017; Vaccarino et al., 2007). Therefore, astrocytes‐based therapies for stroke draw extensive attention of researchers (Li, Liu, Xin, & Chopp, 2014; Trendelenburg & Dirnagl, 2005). Interestingly, in the ischemic brain, reactive astrocytes exhibit the properties of neural stem/progenitor cells (Gotz, Sirko, Beckers, & Irmler, 2015) and transdifferentiate into mature (Duan et al., 2015; Magnusson et al., 2014) and functional (Duan et al., 2015) neurons. In the present study, we further confirmed such transdifferentiation of astrocytes in adult rat brain (Figure 5d). As we have known, only small populations of reactive astrocytes can convert into neurons and most of them become glial scar/astrogliosis. Theoretically, for promotion of brain repair after injury, one of important approaches could be to enhance the capacity of astrocyte‐to‐neuron conversion. PAX6 is an important factor to direct astroglial to neuronal lineages (Heins et al., 2002; Kronenberg et al., 2010).We have reported that ischemic stroke induces expression of PAX6 in reactive astrocytes in adult rat brain, and vascular endothelial growth factor (VEGF) enhances PAX6‐expressed astrocytes and the conversion of astrocyte‐to‐neuron (Shen et al., 2016). The present study directly demonstrate that exogenous expression of PAX6 in the brain can enhance the capability of such conversion (Figure 6e,f).

MiRs function in RNA silencing and post‐translational regulation of gene expression. It has been reported that post‐translation of *Pax6* mRNA is regulated by miRs (Bhinge et al., 2016; de Chevigny et al., 2012). To identify miRs targeting the *Pax6* gene, we performed bioinformatics analysis to screen candidates, which revealed that miR‐365, miR‐7 and miR‐129 have potential binding sites on the 3′‐UTR (Figure 1a). We found that miR‐365, but not miR‐7 or miR‐129, inhibited PAX6 expression in cultured astrocytes (Figure 1c,d). However, previous studies have reported that miR‐7 suppresses PAX6 expression in mouse and human (de Chevigny et al., 2012; Latreille et al., 2014; Li et al., 2014; Needhamsen, White, Giles, Dunlop, & Thomas, 2014). This discrepancy might be caused by species variation. Moreover, we observed an increase in miR‐365 and a decrease in miR‐7 and miR‐129 in the ischemic brain and hypoxic cultured cells (Figure 1e,f). Our findings are consistent with a previous report showing a decrease in miR‐7 of the ischemic rat brain (Dharap, Bowen, Place, Li, & Vemuganti, 2009). Besides, the expression relationship of miR‐365 and PAX6 was negative correlation (Figure 1f,g). Taken together, we found that miR‐365 was increased in the ischemic brain and inhibited PAX6 expression in the astrocytes. Therefore, we focused on miR‐365 for further *in vivo* investigations.

MiR‐365 plays roles in the initiation and development of cancers by repressing bcl‐2 and cyclin D1/cdc25A expression (Guo et al., 2013; Nie et al., 2012). In addition, miR‐365 participates in morphine tolerance and nociceptive behaviors (Pan et al., 2016; Wang et al., 2016). Moreover, miR‐365 is upregulated in the spinal cord of rats with amyotrophic lateral sclerosis and in the hippocampus of epileptic rats (Parisi et al., 2013; Sun et al., 2013). However, the significance of this upregulation in the CNS remains unclear, although it has been reported that miR‐365 increases apoptosis and inflammatory response (Qin et al., 2011; Yang et al., 2016). In the present study we clearly demonstrate that ischemia upregulates miR‐365 (Figure 1e,f). An increase in endogenous miR‐365 levels in the ischemic brain could aggravate neuronal damage because knockdown of miR‐365 by its antagomir significantly reduced neurological deficits and ischemic infarct volume, and enhanced stroke‐induced neurogenesis (Figure 8). Indeed, upregulation of miR‐365 with its agomir had the opposite effects. Collectively, our results demonstrate that, in the ischemic brain, upregulation of miR‐365 is detrimental to brain repair. Conversely, downregulation of miR‐365 is beneficial. We speculate that the increase in infarct volume induced by miR‐365 might be related to a reduction in neurogenesis. Although miR‐365 has been shown to repress bcl‐2 expression in cancer cells and human umbilical vein endothelial cells (HUVECs; Nie et al., 2012; Qin et al., 2011), we still need to determine whether miR‐365 would modulate bcl‐2 expression in adult rat brain after ischemic stroke. More interestingly, our results clearly indicate that miR‐365 directly targets the *Pax6* 3′‐UTR (Figure 2b). Upregulation and downregulation of miR‐365 respectively decreased and increased PAX6 expression *in vitro* (Figure 2d–f) and *in vivo* (Figure 3, d‐f). Moreover, miR‐365 effectively inhibited the expression of endogenous PAX6, but it could not suppress the expression of exogenous lentiviral PAX6, which containing the *Pax6* CDS without the 3′‐UTR (Figure 7a,b). These results clearly demonstrate that miR‐365 inhibits PAX6 expression by targeting the 3′‐UTR.

PAX6 is a pro‐neurogenic transcription factor and highly expresses in reactive astrocytes following ischemia (Duan et al., 2015; Steliga et al., 2013), and it plays a key role in the conversion of astrocytes into neurons (Buffo et al., 2005; Heins et al., 2002; Kronenberg et al., 2010). In the ischemic brain, reactive astrocytes can be driven to reprogram and transdifferentiate into mature and functional neurons (Duan et al., 2015), and this process is inhibited by notch (Magnusson et al., 2014) and enhanced by VEGF (Shen et al., 2016). Here, we found that miR‐365 reduced the conversion of astrocytes into mature neurons by targeting *Pax6* and deteriorated ischemic brain injury. In contrast, miR‐365 knockdown effectively increased PAX6 expression in reactive astrocytes, indicated as an increase in GFAP^+^/PAX6^+^ cells (Figure 4a,b), and promoted the conversion of astrocyte into neuron, evidenced by double labeling for GFP and NeuN (Figure 5d,e). In consistent with a previous study (Kronenberg et al., 2010), exogenous PAX6 expression promoted the reprogramming of astrocytes towards neuronal differentiation. Remarkably, we also noticed that a portion of reprogrammed astrocytes could develop into mature neurons (Figure 6e,f). Moreover, overexpression of PAX6 in the brain abolished the inhibitory effect of miR‐365 on the astrocyte‐to‐neuron conversion (Figure 7e,f). Collectively, our study suggests that increase of endogenous miR‐365 in the brain enlarges infarct volume probably by inhibiting PAX6‐mediated astrocyte‐to‐neuron conversion. Conversely, knockdown of miR‐365 enhances the capacity of brain repair probably via promotion of PAX6‐mediated astrocyte‐to‐neuron conversion. Our findings provide a novel insight into the mechanisms by which miRs regulate neurogenesis.

Recent findings have indicated that, in the ischemic brain, astrocytes participate in the remodeling of neurovascular units (Pan, Mao, & Sun, 2017; Shen et al., 2016), which is an essential structure for functional brain repair (Lo, Dalkara, & Moskowitz, 2003; Lok et al., 2007). The present study reveals that miR‐365 involves in this remodeling process. Knockdown of endogenous miR‐365 or overexpression of PAX6 in the ischemic brain are favorable for the conversion of reactive astrocytes into neurons. It has been reported that these new neurons derived from astrocytes contribute to the reestablishment of functional neural circuitry (Duan et al., 2015). Therefore, both miR‐365 antagomir and PAX6 may become potential approaches to improve remodeling of astrocyte‐neuron networks, a part of neurovascular units.

In summary, the current results demonstrate that the miR‐365–PAX6 system regulates the conversion of astrocytes into neurons in the ischemic brain. Both exogenous expression of PAX6 and administration of miR‐365 antagomir are likely to promote brain repair by enhancing neurogenesis in adult mammalian brain following ischemic injury. Our results suggest that miRs can regulate reactive astrocytes reprogramming and conversion into neurons in adult brain by targeting their specific transcription factors, which may help us to find new therapeutic targets for cerebral ischemic stroke.
